# Conformation and Cross-Protection in Group B Streptococcus Serotype III and *Streptococcus pneumoniae* Serotype 14: A Molecular Modeling Study

**DOI:** 10.3390/ph12010028

**Published:** 2019-02-13

**Authors:** Michelle M. Kuttel, Neil Ravenscroft

**Affiliations:** 1 Department of Computer Science, University of Cape Town, Cape Town 7701, South Africa; 2 Department of Chemistry, University of Cape Town, Cape Town 7701, South Africa; neil.ravenscroft@uct.ac.za

**Keywords:** capsular polysaccharide, carbohydrate antigen, molecular modeling, Group B Streptococcus, *Streptococcus pneumoniae*, conjugate vaccines

## Abstract

Although the branched capsular polysaccharides of *Streptococcus agalactiae* serotype III (GBSIII PS) and *Streptococcus pneumoniae* serotype 14 (Pn14 PS) differ only in the addition of a terminal sialic acid on the GBSIII PS side chains, these very similar polysaccharides are immunogenically distinct. Our simulations of GBSIII PS, Pn14 PS and the unbranched backbone polysaccharide provide a conformational rationale for the different antigenic epitopes identified for these PS. We find that side chains stabilize the proximal βdGlc(1→6)βdGlcNAc backbone linkage, restricting rotation and creating a well-defined conformational epitope at the branch point. This agrees with the glycotope structure recognized by an anti-GBSIII PS functional monoclonal antibody. We find the same dominant solution conformation for GBSIII and Pn14 PS: aside from the branch point, the backbone is very flexible with a “zig-zag” conformational habit, rather than the helix previously proposed for GBSIII PS. This suggests a common strategy for bacterial evasion of the host immune system: a flexible backbone that is less perceptible to the immune system, combined with conformationally-defined branch points presenting human-mimic epitopes. This work demonstrates how small structural features such as side chains can alter the conformation of a polysaccharide by restricting rotation around backbone linkages.

## 1. Introduction

The bacterium *Streptococcus agalactiae*, usually termed Group B Streptococcus, is a primary cause of neonatal sepsis and meningitis, particularly in infants born to carriers of the pathogen. The *Streptococcus pneumoniae* bacterium is another common cause of serious infections in young infants, including meningitis and pneumonia. Ten serotypes of Group B Streptococcus have been characterized, of which serotype III (GBSIII) is currently the most prevalent [1]. Over 90 serotypes of *Streptococcus pneumoniae* have been identified, with serotype 14 (Pn14) being the most common cause of invasive pneumococcal disease in children prior to the introduction of conjugate vaccines [2].

Both of these gram-positive bacteria are encapsulated by polysaccharides that vary in structure according to bacterial serotype and are essential for bacterial virulence: vaccination with carbohydrate-protein conjugates can provide effective serotype-specific protection. The Pn14 capsular polysaccharide (PS) is a component of all licensed conjugate vaccines since the introduction of the 7-valent Prevenar vaccine. GBSIII PS is present in a trivalent conjugate vaccine targeting serotypes Ia, Ib, and III that has completed phase-2 trials [3,4] and a hexavalent vaccine currently in clinical trials.

The similarity of the branched GBSIII PS and Pn14 PS has long been of interest [5]: they are identical except that the GBSIII PS carries a terminal α(2→3)-linked sialic acid (αdNeu5Ac) on the galactose side chain. The Pn14 PS thus has a four-residue repeat unit (RU) and the GBSIII PS a five-residue RU, as follows.
Pn14 PS:→6)[βdGal*p*(1→4)]βdGlc*p*NAc(1→3)βdGal*p*(1→4)βdGlc*p*(1→GBSIII PS:→6) [α**d****Neu5Ac(2→3)**βdGal*p*(1→4)]βdGlc*p*NAc(1→3)βdGal*p*(1→4)βdGlc*p*(1→

This structural similarity of the GBSIII PS and Pn14 PS raises the possibility that type-specific antibodies induced by one of these organisms might protect against disease caused by the other, a phenomenon referred to as cross-protection. Indeed, vaccination with GBSIII PS has been shown to raise two types of anti-carbohydrate antibody: a major population recognising the native PS but not the desialylated PS (equivalent to Pn14 PS) and a minor population that cross-reacts with Pn14 PS. However, the converse has not been found to be true: antibodies elicited by Pn14 PS are not protective against GBSIII bacteria, although desialylation of the GBSIII PS significantly increases the cross-reactivity with Pn14 antibodies [6,7]. Evidence for serotype cross-protection is necessarily indirect and complicated by the fact that cross-reaction of a PS with antibody raised by a different PS does not reliably predict cross-protection: vaccination raises families of antibodies against various PS epitopes, not all of which are of high avidity and thus effective opsonophagocytic (killing) antibodies. Indeed, effective cross-protection between GBSIII and Pn14 has not been demonstrated: antibodies elicited by Pn14 PS (desialylated GBSIII PS) are not protective against GBSIII bacteria and there is considerable evidence that the presence of the terminal sialic acid residue is essential for the elicitation of protective antibodies against GBSIII PS [6,7,8,9].

**Figure 1:**
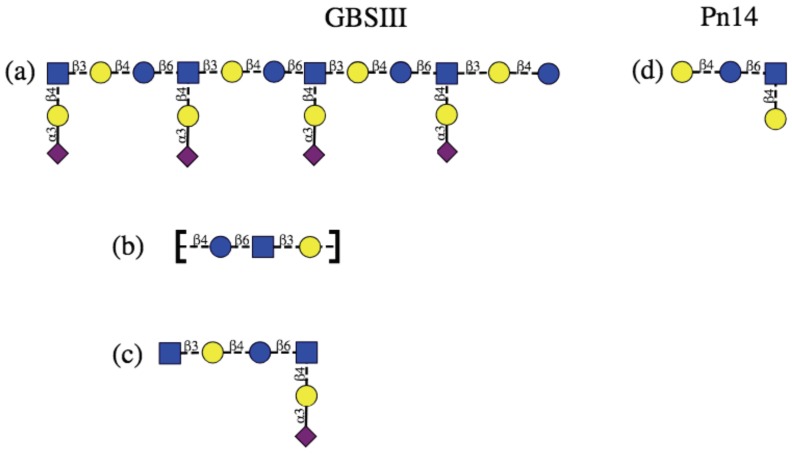
Schematic representation of the protective epitopes previously identified for GBS PS (left column) and Pn14 PS (right column). GBSIII: (**a**) 3–4 RU helical conformational epitope postulated for GBSIII PS [10,11]; (**b**) the linear backbone epitope identified from fragment binding to GBSIII antibodies [14] and (**c**) a 6-residue epitope identified from a DP2-Fab crystal structure [13]. Pn14: (**d**) the tetrasaccharide epitope first identifed by Safari et al. [7,14] and then by Kurbatova et al. [15] from antibody studies. Structures are depicted using the ESN symbol set [16] with yellow circle: Gal, blue circle: Glc, blue square: GlcNAc, purple diamond: Neu5Ac.

The native PS produced by GBSIII and Pn14 contains between 50 and 300 RU, which is far longer that the epitope bound by an antibody. There has been some effort expended into identification of the minimal epitope for both GBSIII PS and Pn14 PS, with some conflicting results (Figure 1). Originally, on the basis of NMR measurements and molecular modeling, a long 3–4 RU helical conformational epitope for GBSIII PS was proposed (Figure 1a), with the Pn14 PS being comparatively flexible and disordered. The antigenic differences between GBSIII and Pn14 were thus originally attributed to significantly different PS conformations (and hence conformational epitopes), rather than direct interaction of the antibody with the sialic acid side chain in GBSIII PS [9,10,11,12]. The hypothesis was that anti-GBSIII antibodies bind the helical GBSIII PS backbone (stabilized by the sialic acid residues on the exterior surface of the helix) and not the sialylated side chain, with a 3 to 4 RU epitope necessary for raising protective antibodies.

However, this hypothesis is challenged by later work that provides evidence that a helical conformational epitope of GBSIII PS is not required for antigen recognition and that the sialic acid participates directly in antibody recognition of GBSIII. Safari et al. investigated the epitope specificity of GBSIII and showed that human anti-GBSIII PS antibodies recognized the linear backbone epitope common to Pn14 PS and GBSIII PS: -Glc-GlcNAc-Gal- (Figure 1b) [7]. However, although conjugates of linear oligosaccharides of GBSIII PS (such as Gal-Glc-GlcNAc, Glc-GlcNAc-Gal, and GlcNAc-Gal-Glc) did evoke specific oligosaccharide antibodies in mice, these antibodies bound neither native nor desialylated GBSIII PS. Therefore, it was assumed that they are too small or flexible to raise antibody and longer chain lengths exhibiting this epitope are required. Furthermore, the recent elucidation of the crystal structure of a short six-sugar GBSIII epitope in complex with a functional antibody showed a branched hexasaccharide functional epitope with sialic acid participating directly in antigen binding [13]. Identification of a short epitope for GBSIII is valuable information for the development of synthetic vaccines, due to the difficulty and cost associated with synthesis of longer oligosaccharides.

Effective short, branched epitopes have also been identified for the Pn14 PS: Safari et al. demonstrated that one RU of the Pn14 PS (Figure 1c) is essential and sufficient for inducing protective Pn14-specific antibodies: the presence of the trisaccharide branch point in an epitope is crucial for anti-Pn14 antibody recognition and the extra galactose contributes to the immunogenicity of the epitope [14]. Kurbatova et al. recently identified a similar branched tetrasaccharide as the most effective epitope for Pn14 PS [15]. In contrast, linear Pn14 PS fragments were found to be completely ineffective: none of a range of short linear epitopes of the PS backbone were recognized by Pn14 antibodies [7]. Only branched fragments containing the Gal-Glc-(Gal-)GlcNAc moiety were found to provide significant protection.

Identification of the capsular polysaccharide epitopes recognized by protective antibodies (or glycotopes) and their conformation is crucial for understanding the affinity and specificity of carbohydrate-antibody interactions and, ultimately the cross-protection mechanisms. Furthermore, identification of the conformational effect of side chains on PS conformation may usefully inform optimal antigen design and the development of effective conjugate vaccines. However, as direct experimental evidence of the key PS epitopes is difficult to obtain, systematic molecular modeling protocols have been developed to provide a theoretical estimate of carbohydrate conformation and dynamics [17]. In the last decade, a considerable improvement in both carbohydrate force fields [18,19,20,21] and computer hardware has facilitated far larger, longer and more accurate computer simulations of polysaccharides than was possible when the previous simulations of GBSIII/Pn14 PS were performed. Therefore, we considered it timely to embark on more extensive modeling of these capsular polysaccharides. Our aim is to shed light on the remaining unanswered questions on the conformations of the GBSIII and Pn14 PS, including the following. Does the GBSIII PS have a helical conformation? Are the conformation and dynamics of the GBSIII PS significantly different to Pn14 PS? What are the likely minimal epitopes for these two PS? Why are some fragments more effective antigens than others?

To answer these questions, we compare the solution conformation and dynamics of the GBSIII and Pn14 PS, as well as the corresponding unbranched saccharide, to determine the effects of the side chains on the polysaccharide backbone conformation. We ran long simulations of 1 μs, an order of magnitude more than the previous simulations of 50 ns.

We do not consider O-acetylation of the terminal αdNeu5Ac sialic acid residues [22], as O-acetylation was not found to be necessary for elicitation of functional antibodies against GBSIII [23]. We find that contrary to previous simulations, the polysaccharide backbone dynamics is almost identical in the GBSIII and Pn14 polysaccharides. The backbone is not helical, but rather has a highly flexible zig-zag conformation, whereas the branch points are relatively inflexible with well-defined conformational epitopes. In contrast, the unbranched PS is highly flexible and conformationally varied. Our results are supported by NMR NOESY experiments performed on the GBSIII polysaccharide.

## 2. Materials and Methods

Our established systematic approach to modeling of polysaccharide antigens involves first determining the preferred conformations of each of the glycosidic linkages in the polysaccharide by calculation of the ϕ, ψ potential of mean force (PMF) for the corresponding disaccharides and then progresses to molecular dynamics simulations of three- and six-RU oligosaccharides in aqueous solution to establish the preferred conformations and dynamics of the carbohydrate chains [24,25,26].

### 2.1. Disaccharide PMF Calculations

We identified the preferred conformations of the each of the glycosidic linkages in isolation by calculation of the potential of mean force (PMF) for rotation about the ϕ and ψ dihedral angles. PMFs were calculated using the metadynamics [27] routine incorporated into NAMD [28] with the glycosidic linkage torsion angles used as collective variables. For the three-bond (1→6) linkages, we calculated a two-dimensional PMF as a function of ϕ and ψ only, allowing the ω dihedral to rotate freely. All PMF surfaces were calculated in gas-phase, except for the charged αdNeu5NAc(2→3)βdGal*p* disaccharide, which required simulation in explicit aqueous solution with a neutralizing counter-ion. Gas phase PMFs for uncharged disaccharides have been demonstrated to be a reasonable approximation to solution PMF in a polysaccharide [25,29,30].

### 2.2. Molecular Dynamics Simulations

All simulations were performed with the NAMD molecular dynamics program [28] version 2.12 (employing NAMD CUDA extensions for calculation of long-range electrostatics and nonbonded forces on graphics processing units [31]). Carbohydrates were modeled with the CHARMM36 additive force field for carbohydrates [19,32] and water was simulated with the TIP3P model [33].

Initial configurations of three-repeat (3 RU) and six-repeat unit (6 RU) oligosaccharides for GBSIII and Pn14 PS were built using our in-house CarbBuilder software [34,35] which employs the psfgen tool to create “protein structure” (psf) files for modeling with the CHARMM force field and the NAMD molecular dynamics program. These initial oligosaccharide structures were optimized through 20,000 steps of standard NAMD minimization in vacuum and then solvated (using the *solvate* plugin to the Visual Molecular Dynamics (VMD) [36] analysis package) in a periodic cubic unit cell with randomly distributed sodium ions to electrostatically neutralize the system.

All MD simulations were preceded by a 30,000 step minimization phase, with a temperature control and equilibration regime involving 10 K temperature reassignments from 10 K culminating in a maximum temperature of 300 K. Equations of motion were integrated using a Leap-Frog Verlet integrator with a step size of 1 fs and periodic boundary conditions. Simulations were performed under isothermal-isobaric (nPT) conditions at 300 K maintained using a Langevin piston barostat [37] and a Nose-Hoover [38,39] thermostat.

Long-range electrostatic interactions were treated using particle mesh Ewald (PME) summation, with κ=0.20 Å${}^{-1}$ and 1 Å PME grid spacing. Non-bonded interactions were truncated with a switching function applied between 12.0 and 15.0 Å to groups with integer charge. The 1–4 interactions were not scaled, in accordance with the CHARMM force field recommendations.

Each metadynamics simulation comprised a 1500 ns MD simulation, with a Gaussian hill height of 0.5 and width of 2.5 degrees. Structures were collected at intervals of 250 ps for analysis. For the solution simulation, the αdNeu5NAc(2→3)βdGal*p* disaccharide was placed in the center of a cubic box with sides of 30 Å. The box was filled with approximately 2500 water molecules and a single Na${}^{+}$ counter ion.

The 3RU strands were placed in the center of a cubic water box with sides of 60 Å, while the 6RU strands employed a box of length 80 Å. The Pn14 strands were solvated with 6810 (3RU) and 16,313 (6 RU) water molecules, while the GBSIII strands used 6790 (3 RU) and 16,279 (6 RU) water molecules. The GBSIII systems were neutralized with 3 (3 RU) and 6 (6 RU) Na${}^{+}$ ions. In each case, the system was equilibrated 0.03 ns with a cycled temperature increase from 0 K to 300 K in 10 K increments with each cycle commencing with a 10,000 step energy minimization followed by a 0.001 ns MD simulation at the specified temperature until 300 K. The 3 RU MD simulations ran for 250 ns and the 6 RU simulations ran for 1 μs.

### 2.3. Data Analysis

In this work, two-bond (1→X) glycosidic linkages are defined by the torsion angles ϕ = $H1^{\prime }$–$C1^{\prime }$–OX–CX and ψ = $C1^{\prime }$–OX–CX–HX. The (2→3) glycosidic linkages use ϕ = $C1^{\prime }$–$C2^{\prime }$–O3–C3 and ψ = $C2^{\prime }$–O3–C3–H3. These definitions for ϕ and ψ are analogous to $\phi_{H}$ and $\psi_{H}$ in IUPAC convention. For the (1→6) glycosidic linkage, the three dihedral angles are defined as ϕ = $H1^{\prime }$–$C1^{\prime }$–O6–C6, ψ = $C1^{\prime }$–O6–C6–C5 and ω = O6–C6–C5–O5.

Analysis of the simulations used time series frames 25 ps apart, discarding the first 100 ns as equilibration. Molecular conformations extracted from the MD simulations were depicted with VMD, where necessary using the PaperChain visualization algorithm for carbohydrates [40] to highlight the hexose rings. Dihedral angles from the simulations and the DP2-Fab complex crystal structure (PDB ID code 5M63) were extracted using VMD’s Tcl scripting interface and statistical values calculated with in-house Python scripts. The DP2 fragment comprises RU2 with a terminal 2,5-anhydro-d-Man. The GBSIII 6RU simulation conformations and the DP2-Fab complex crystal structure were aligned for comparison on the ring atoms of the αdNeu5NAc(2→3)βdGal*p*(1→4)βdGlc*p*NAc branch point.

Conformations from both 3RU trajectories were clustered using VMD’s internal *measure cluster* command to calculate clusters according to the quality threshold algorithm [41]. Frames were aligned on the GlcNAc residues in RU1 to RU5 of the 6RU chains. Clustering was then performed with a cut-off of 7 Å on an RMSD fit to the atoms in the backbone residues of RU1 to RU5.

### 2.4. NMR Analysis

The GBSIII polysaccharide sample (10 mg) was lyophilized and exchanged twice with 99.9% D${}_{2}$O (Sigma Aldrich, Pty. Ltd., Johannesburg, South Africa), then dissolved in 600 μL of D${}_{2}$O and introduced into a 5 mm NMR tube for data acquisition. 1D ${}^{1}$H and ${}^{13}$C and 2D, COSY, TOCSY, NOESY, HSQC, HMBC and hybrid HSQC-TOCSY and HSQC-NOESY spectra were obtained using a Bruker Avance III 600 MHz NMR spectrometer (Bruker BioSpin AG, Fällanden, Switzerland) equipped with a BBO Prodigy cryoprobe and processed using standard Bruker software (Topspin 3.2). The probe temperature was set at 343 K. The 2D TOCSY experiment was performed using a mixing time of 180 ms and the 1D variants using a mixing time of 200 ms. The 2D NOESY experiment was performed using a mixing times of 300 and 500 ms and the 1D variants using mixing times of 300, 400 and 500 ms. The ${}^{1}$H-${}^{13}$C HSQC and HMBC experiments were optimized for J = 145 Hz and 8 Hz, and the HSQC-TOCSY and HSQC-NOESY experiments were recorded using mixing times of 120 and 250 ms, respectively. 2D experiments were recorded using non-uniform sampling: 50% for homonuclear and 25% for heteronuclear experiments. Spectra were referenced relative to the H3ax/C3 signal of terminal sialic acid: ${}^{1}$H at 1.79 ppm and ${}^{13}$C at 40.68 ppm [42].

## 3. Results

Our 1 μs simulations of 6 RU show that polysaccharides with the →6)βdGlc*p*NAc(1→3)βdGal*p*(1→4)βdGlc*p*(1→ backbone are all extremely flexible, with none of the well-defined conformations we have found in other, linear bacterial polysaccharides [24,25]. However, a closer inspection of the range of motion for GBSIII PS, Pn14 PS and the unbranched backbone PS reveal a common dominant conformational habit, as well as significant differences in flexibility.

### 3.1. PS Chain Conformations

**Figure 2:**
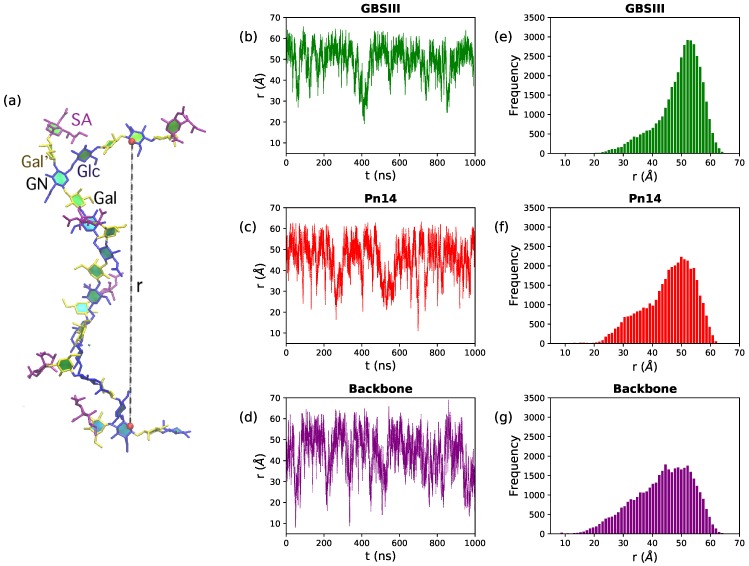
Polysaccharide end-to-end distance distributions for the 6RU PS. (**a**) The molecular end-to-end distance, *r*, is defined as the distance between O5 atoms in GlcNAc residues in RU1 and RU6, here shown on the GBSIII PS. The center column shows the *r* time series for (**b**) GBSIII PS, (**c**) Pn14 PS and (**d**) unbranched backbone PS. The corresponding distance distributions are in the rightmost column for (**e**) GBSIII PS, (**f**) Pn14 PS and (**g**) the unbranched backbone PS.

In Figure 2, we quantify range of motion for these very flexible polysaccharides with the simple end-to-end distance, *r*, measured between the GlcNAc O5 atoms in repeat units RU1 and RU6 (illustrated on a sample conformation in Figure 2a). Comparison of the time series (Figure 2, center column) and distribution (right column) of *r* shows that GBSIII PS is the most conformationally defined, and the unbranched polysaccharide backbone the most flexible, of the three molecules. Only 12% of the GBSIII PS conformations have r<40 Å, as compared to 25% for Pn14 and 33% for the unbranched backbone. Overall, the unbranched backbone is less extended (more globular) than the branched polysaccharides and shows more rapid conformational transitions (compare time series plots in Figure 2b–d). The corresponding mean squared end-to-end distances for the simulations reflect this trend: 2505 Å (GBSIII), 2157 Å (Pn14) and 1993 Å (backbone).

**Figure 3:**
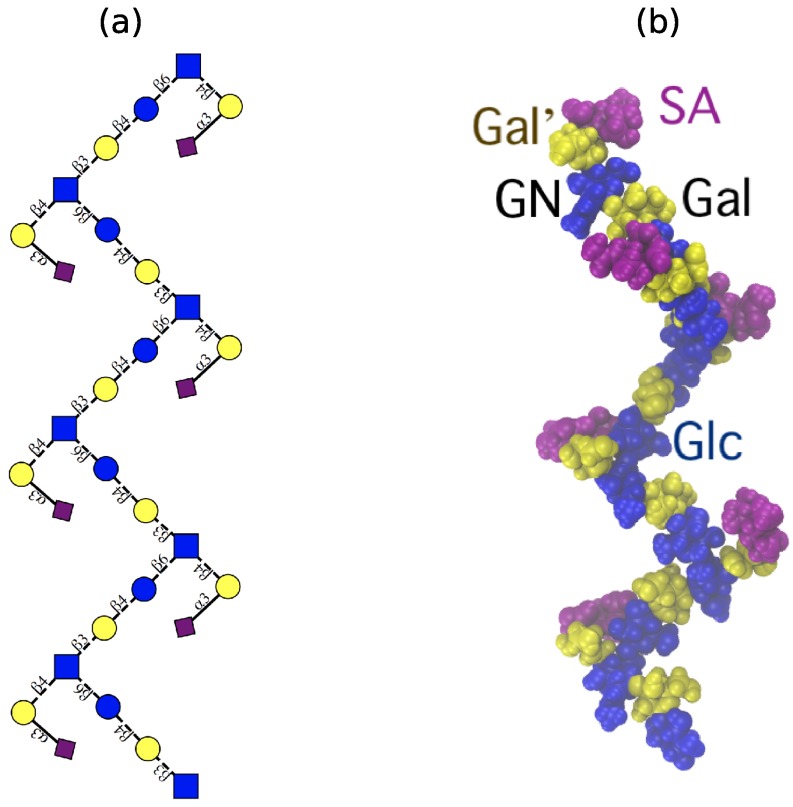
The ‘zig-zag’ conformational habit of GBSIII and Pn14 PS. (**a**) Schematic representation with the ESN symbol set [16] (yellow circle: Gal, blue circle: Glc, blue square: GlcNAc, purple diamond: Neu5Ac) and (**b**) a representative 6RU GBSIII PS simulation snapshot. Residues are highlighted as follows: Glc and GlcNAc: blue; Gal: yellow; sialic acid: purple.

Although flexible, the GBSIII PS and Pn14 PS have a similar dominant overall conformation with 48<r<58 Å. Here the PS backbone has an overall “zig-zag” arrangement, bending at the 1→6 linkage, with the side chains exposed at the branch points, as shown in the GBSIII schematic in Figure 3a and the sample conformation in Figure 3b.

**Figure 4:**
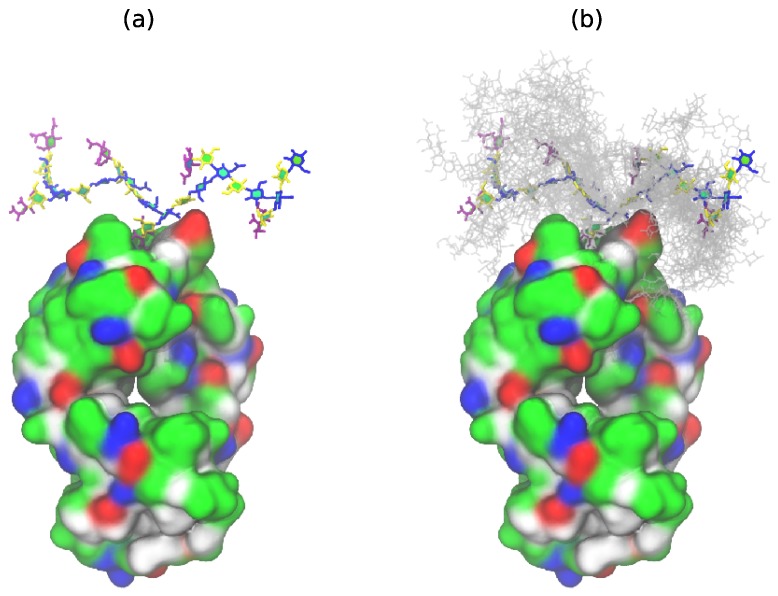
GBSIII 6RU structures superimposed on the bound branch point of the DP2-FAb crystal structure from Carboni et al. [13] (PDB ID code 5M63): (**a**) a single representative structure and (**b**) superimposed conformational snapshots at 12.5 ns intervals (with the first 125 ns discarded as equilibration).

This conformation is in very good agreement with the new antibody binding model recently proposed for GBSIII PS (see Figure 5 in Carboni et al. [13]). Figure 4 shows the GBSIII PS conformations from our 6RU simulation superimposed on the solved crystal structure for DP2-Fab. The zig-zag conformation of the backbone exposes the sialic acid side chain for binding (Figure 4a) with the flexible backbone held well away from the antibody over the course of the simulation (Figure 4b). Conversely, we find no evidence in our simulations to support the helical model for GBSIII PS previously proposed [10,11]. Further, in the zig-zag conformation, the polysaccharide backbone is relatively inaccessible to antibody binding.

However, although the 6RU backbone shows the same dominant conformation for GBSIII PS, Pn14 PS and the unbranched backbone PS, they have a significant difference in flexibility. Clustering of the simulation conformations shows that, while GBSIII PS is in this general zig-zag conformational family for 85% of the simulation, Pn14 PS is more flexible and is in a zig-zag for 78% of the simulations, moving occasionally into alternative, more bent conformations (with smaller *r*). The unbranched backbone polysaccharide is the most disorganised, with the zig-zag appearing for 66% of the simulation and showing significant sub-populations of globular conformations (with *r* < 25 Å).

**Figure A1:**
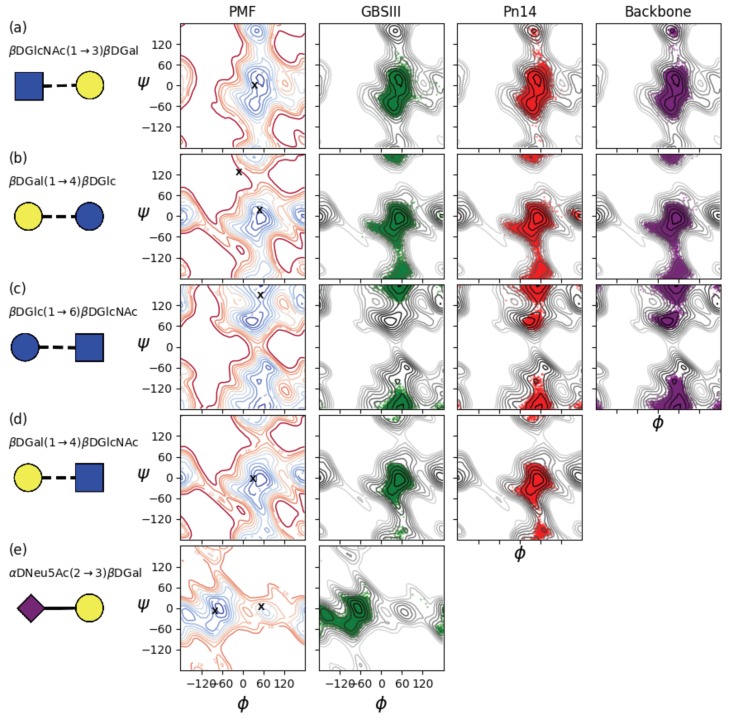
The left column shows contoured disaccharide ϕ, ψ PMF surfaces for (**a**) βdGlcNAc(1→3)βdGal, (**b**) βdGal(1→4)βdGlc, (**c**) βdGlc(1→6)βdGlcNAc, (**d**) βdGal(1→4)βdGlcNAc and (**e**) αdNeu5Ac(2→3)βdGal. The PMF for the charged αdNeu5Ac(2→3)βdGal disaccharide is in solution, all others are gas-phase. Contours are drawn at intervals of 1 kcal·mol${}^{-1}$ to a maximum of 12 kcal·mol${}^{-1}$ and ‘X’ markers indicate dihedral angle values from the six-sugar GBSIII epitope in complex with Fab crystal structure [13]. Scatter plots of the corresponding two central linkages in the 6RU oligosaccharide strands for the last 900 ns of simulation time are superimposed on the PMFs for 6RU of GBSIII (column 2, green), Pn14 (column 3, red) and the unbranched backbone (right column, purple).

The source of the conformational differences between the polysaccharides is a conformational constraint on the βdGlc(1→6)[βdGal(1→4)]βdGlcNAc linkage in GBSIII PS and, to a lesser extent, in Pn14 PS. The conformation and flexibility of the other backbone linkages (βdGlc*p*NAc(1→3)βdGal*p* and βdGal*p*(1→4)βdGlc*p*) is very similar in all three polysaccharides—see a detailed analysis in Appendix A, particularly Figure A1. However, for the βdGlc*p*(1→6)βdGlc*p*NAc linkage, proximity of the side chains impose a restriction on rotation, as follows.

### 3.2. Conformations of the 1→6 Linkage

**Figure 5:**
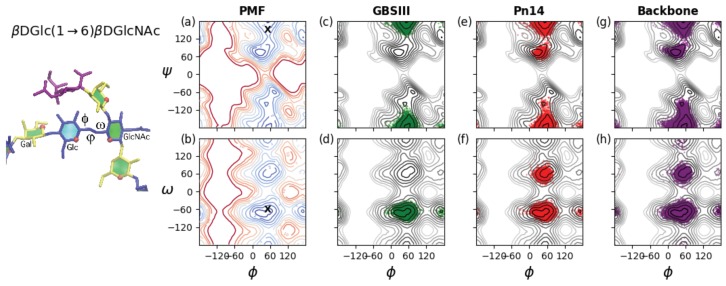
Rotation of the three-bond βdGlc(1→6)βdGlcNAc linkages in GBSIII, Pn14 and the unbranched backbone PS. The ϕ, ψ and ω angles describing the orientation of this linkage are labeled on the GBSIII fragment shown on the left. Contoured 2D (**a**) ϕ, ψ PMF and (**b**) the ϕ, ω PMF surfaces for a βdGlc(1→6)βdGlcNAc disaccharide in the gas phase illustrate the range of motion possible for an unrestrained linkage. Contours are drawn at intervals of 1 kcal·mol${}^{-1}$ to a maximum of 12 kcal·mol${}^{-1}$ and ‘X’ markers indicate dihedral angle values from the six-sugar GBSIII epitope identified in the DP2-Fab crystal structure [13]. The range of motion actually explored by the 6RU oligosaccharides is demonstrated with time series scatter plots of the two central βdGlc(1→6)βdGlcNAc linkages in the 6RU strands superimposed on the PMFs as follows: (c) GBSIII ϕ, ψ; (d) GBSIII ϕ, ω; (e) Pn14 ϕ, ψ; (f) Pn14 ϕ, ω; (g) unbranched backbone ϕ, ψ; and (f) unbranched backbone ϕ, ω.

Figure 5 provides a comparison of the flexibility of the three-bond glycosidic linkage in a βdGlc*p*(1→6)βdGlc*p*NAc disaccharide and the GBSIII PS, Pn14 PS and unbranched backbone PS. The three dihedral angles describing rotation about the three bonds in the (1→6) glycosidic linkage are here defined as ϕ = $H1^{\prime }$–$C1^{\prime }$–O6–C6, ψ = $C1^{\prime }$–O6–C6–C5 and ω = O6–C6–C5–O5. These dihedrals are labeled on the GBSIII fragment in Figure 5, left. A PMF energy surface for the (1→6) glycosidic linkage has, therefore, three dimensions. However, for ease of visualization and comprehension, we show only 2D projections of the 3D volume: the ϕ, ψ PMF (Figure 5a) and the ϕ, ω PMF surface (Figure 5b) for a βdGlc(1→6)βdGlcNAc disaccharide in the gas phase. These PMF surfaces illustrate the range of motion possible for an unrestrained linkage. The ϕ, ψ PMF in Figure 5a reveals that the central bond in the linkage described by the ψ dihedral is relatively flexible, with multiple minima within the 2 kcal·mol${}^{-1}$ contours: a global minimum conformation at ψ = 71${}^{\circ }$, a secondary anti minimum conformation at ψ = −179${}^{\circ }$ and a tertiary minimum in the ψ = −60 region. In contrast, rotation about the ϕ dihedral is much more constrained, with a broad global minimum around 0${}^{\circ }$ < ϕ < 75${}^{\circ }$ and a narrow secondary well at ϕ = 160${}^{\circ }$. The ϕ, ω PMF surface in Figure 5b confirms these minima for ϕ and shows the expected three minima for the ω dihedral, which are by convention termed gg (ω ≈ −60${}^{\circ }$), gt (ω ≈ 60${}^{\circ }$) and tgω≈ 180${}^{\circ }$). Glucopyranosides are expected to be primarily in the gg conformation, with the gg:gt:tg population ratios approximately 6:4:0 [43].

As a comparison to the unrestrained disaccharide, the range of motion actually explored during the simulations of the 6RU oligosaccharides is revealed by the corresponding dihedral time series scatter plots superimposed on the disaccharide PMFs. The restriction of the βdGlc(1→6)βdGlcNAc linkage in GBSIII is apparent in the time series scatterplots for the 6RU GBSIII oligosaccharide strand (Figure 5, second column). These confirm a single dominant conformer for GBSIII, with average torsion angle values ϕ, ψ, ω = 51${}^{\circ }$, −175${}^{\circ }$, −67${}^{\circ }$ (standard deviations = 10, 11, 8 respectively). These angles are compatible with the GBSIII DP2-Fab complex crystal structure where this linkage has ϕ, ψ, ω = 51${}^{\circ }$, 140${}^{\circ }$, −70${}^{\circ }$ (indicated by ‘X’ on the PMF plots in Figure 5a,b). The central 1→6 linkage in GBSIII PS is in this dominant conformation for 100% of the simulation and the ω dihedrals remain in the low-energy gg conformation for the duration.

The average torsion angle values for Pn14 PS are ϕ, ψ, ω = 50${}^{\circ }$, 180${}^{\circ }$, −62${}^{\circ }$ (standard deviations = 12, 18, 26 respectively). The central βdGlc(1→6)βdGlcNAc linkage is in the dominant conformation (equivalent to the GBSIII PS) for 91% of the simulation. The increase in standard deviation for the ϕ and ω dihedrals relative to GBSIII PS is indicative of increased flexibility and alternative conformations of the ω dihedral, which shows a ratio of approximate 10:1 of gg to gt conformations, with no tg conformations appearing. Therefore, the 1→6 linkage shows a slight increase in flexibility relative to GBSIII PS, which is cumulative with increasing chain length. This reflects the increased constraint on the backbone imposed by the terminal sialic in the GBSIII side chain.

The 1→6 linkage is markedly more flexible in the unbranched backbone saccharide and the zig-zag conformation drops to 86% of the simulation. The average torsion angle values for the unbranched backbone are ϕ, ψ, ω = 50${}^{\circ }$, −179${}^{\circ }$, −69${}^{\circ }$ (standard deviations = 15, 22, 30 respectively). In particular, the ω dihedral has an 8% population of gt conformations and a small population of tg conformations. In addition, both the ϕ and ψ dihedrals show a broader range of rotation.

**Figure A2:**
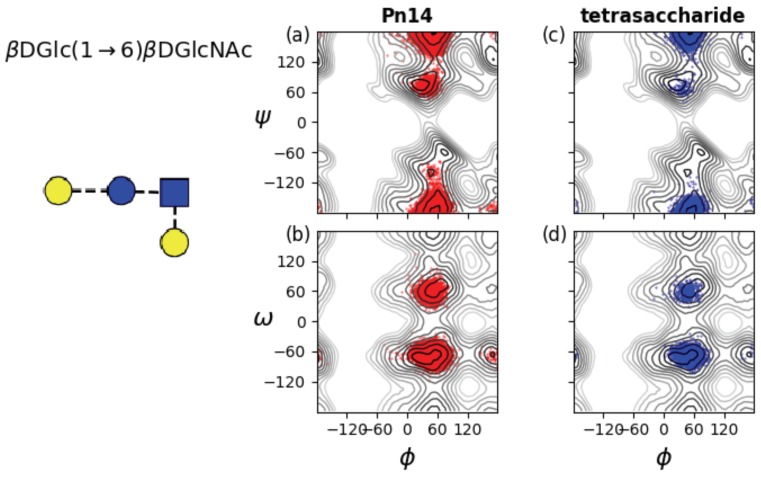
Rotation of the three-bond βdGlc(1→6)βdGlcNAc linkages in Pn14 and the effective tetrasaccharide epitope previously identified (left) [14]. Time series scatter plots are superimposed on the ϕ, ψ PMF (top row) and the ϕ, ω PMF (bottom row) for the two central βdGlc(1→6)βdGlcNAc linkages for: (**a**) Pn14 ϕ, ψ; (**b**) Pn14 ϕ, ω; (**c**) tetrasaccharide ϕ, ψ; and (**d**) tetrasaccharide ϕ, ω.

Interestingly, we saw no effect on conformation and dynamics of increasing chain length. Our simulations of 3RU for GBSIII and Pn14 PS showed the same dihedral populations as the middle linkages in the 6RU simulations (data not shown). Further, we performed a 250 ns simulation of the effective branch epitope previously identified (Figure 1d) [14,15]. This molecule shows the same conformation of the branch point and other dihedrals as Pn14 PS (see Appendix B, Figure A2) and is thus a faithful representation of the branch point in the Pn14 PS.

### 3.3. Inter-Residue Atomic Contacts in GBSIII

In GBSIII PS, interactions between the side chain and the backbone residues stabilize the conformation of the 1→6 linkage. We compare the simulation data with NMR NOESY experiments on the GBSIII PS, focussing on inter-residue NOEs that are diagnostic for close proximity of the side chain to the backbone.

**Figure 6:**
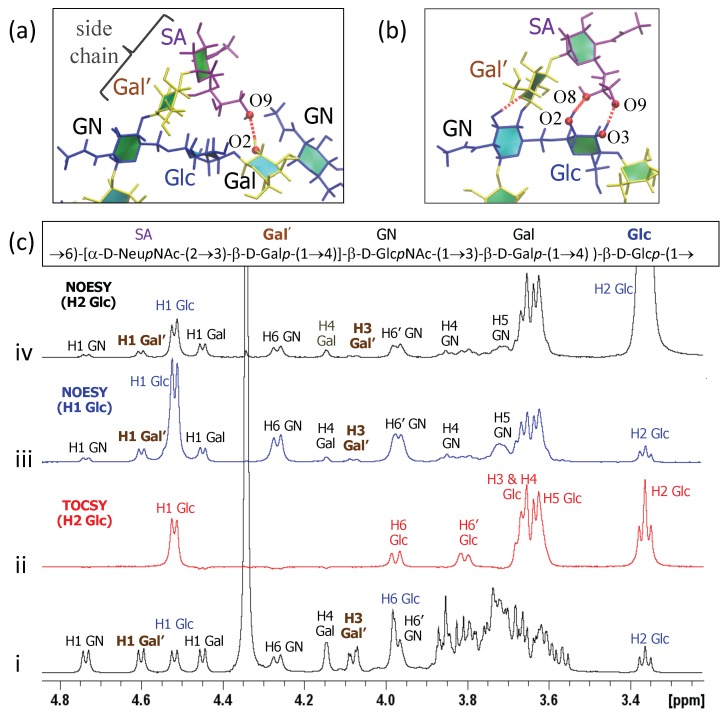
Inter-residue contacts in GBSIII PS. (**a**) Example of SA O9–Gal O2 hydrogen bond between the side chain and the backbone residues. (**b**) Example of simultaneous SA O9–Glc O3 and SA O8–Glc O2 hydrogen bonds. (**c**) Overlay of 1D NMR experiments of GBSIII PS showing (**i**) 1D proton spectrum; (**ii**) 1D TOCSY (200 ms) with irradiation of H2 Glc; (**iii**) 1D NOESY (500 ms) with irradiation of H1 Glc; and (**iv**) 1D NOESY (500 ms) with irradiation of H2 Glc.

In GBSIII PS, hydrogen bonding interactions between the side chain sialic acid (SA) and side chain Gal (Gal’) and the backbone Glc and Gal stabilize the conformation of the 1→6 linkage, as follows. Transient hydrogen bonds occur between the bound SA O9 and backbone Gal O2 (shown in Figure 6a) as well as SA O9 – Glc O3 and SA O8 – Glc O2 hydrogen bonds (occasionally simultaneous, as in Figure 6b). The crystal structure for the GBSIII PS fragment in DP2-Fab shows a close hydrogen bond between SA O9 and and the Glc O6, with distance of 2.8 Å [13]. We do not find this hydrogen bond in our simulations, although the atoms come within 4 Å of each other. This is due to the fact that the sialic acid is in an alternative (higher energy) conformation in the crystal structure—possibly stabilized by interactions with the antibody. The conformation of this sialyl linkage is known to have no fixed standard conformation and to be heavily dependent on the molecular environment [44]. See Appendix A and Figure A1e for more detailed analysis of the sialic acid conformation. Unfortunately, considerable overlap between the signals of the NAc group of SA and GlcNAc precluded investigation of contact between the sialic acid side chain and the backbone in GBSIII, as previously reported [13].

Additional hydrogen bonds occur between the Gal’ O2 and backbone Glc O3 in both the GBSIII PS and Pn14 PS simulations. In this case, NMR signals are better resolved: a series of 1D and 2D NOESY experiments gave key H1 Glc crosspeaks to H4, H5 and both H6s of the neighboring GlcNAc (consistent with the βdGlc(1→6)[βdGal(1→4)]βdGlcNAc linkage) as well as small additional correlations to the main chain repeating unit (Figure 6c(iii)). In particular, peaks for H1 Glc to H1 Gal’ as well as H1 Glc to H3 Gal’ provide evidence for close proximity of the side chain to the backbone and are consistent with our simulations. This is corroborated by 2D NOESY cross peaks, as well as peaks from the well-resolved H2 of Glc at 3.36 ppm to H1 and H3 of Gal’ (Figure 6c(iv)).

## 4. Discussion

This modeling study shows that the polysaccharide backbone conformation is very similar in the GBSIII and Pn14 polysaccharides, albeit with increased flexibility for Pn14 PS. Both GBSIII and Pn14 PS exhibit a constrained branch epitope and a flexible zig-zag backbone. The zig-zag conformation is in remarkable agreement with the binding model for GBSIII PS proposed by Carboni et al. [13]. The βdGlc(1→6)[βdGal(1→4)]βdGlcNAc branch point is a relatively rigid and exposed component of the flexible backbone, and thus a likely site for antibody binding. This stationary branch point is in agreement with preclinical evidence that has indicated that the Pn14 branching element βdGal*p*(1→4)]βdGlc*p*NAc is necessary and sufficient for induction of an effective antibody response [14]. Further, the lack of accessibility of the backbone in GBSIII and Pn14 PS to antibody binding together with increased flexibility and alternative conformations of the unbranched backbone provides a rational for why antibodies to linear oligosaccharide fragments of GBSIII PS (such as Gal-Glc-GlcNAc, Glc-GlcNAc-Gal, and GlcNAc-Gal-Glc) bound neither GBSIII PS nor Pn14 PS [7]. In addition, the lack of extended conformational epitopes supports experimental data that finds short epitopes to be effective for GBSIII and Pn14, epitopes comprising solely their respective branch points and side chain (i.e., βdGlc(1→6)[αdNeu5Ac(2→3)βdGal(1→4)]βdGlcNAc and βdGlc(1→6)[βdGal(1→4)]βdGlcNAc, respectively). This is consistent with the DP2-Fab crystal structure elucidated by Carboni et al.: the binding involves the branch and does not require a conformational epitope [13].

Kurbatova et al. identified a βdGal*p*(1→4)βdGlc*p*(1→6)[βdGal*p*(1→4)]βdGlc*p*NAc tetrasaccharide as the most effective epitope for Pn14 PS, as compared to hexa- and octasaccharides in a mouse model [15]. Our work provides a possible explanation for this: vaccination with this primary epitope could raise a single class of effective antibody, as opposed to a family of antibodies raised by the more flexible hexa- and octasaccharides, thus making this short chain more protective. In contrast, the hexa- and octasaccharides could potentially present epitopes not present in the polysaccharide, as seen in the unbranched backbone.

The flexible zig-zag conformation and stationary branch point were not identified for the Pn14 PS in the earlier study. In contrast to the small 8% population of the gt conformation in Pn14 that we see in these simulations, in the 5RU simulations with the AMBER force field by González-Outeiriño et al. the Pn14 oligosaccharide showed a nearly 50:50 mixture of the gg to gt rotamers, whereas the GBSIII PS remained almost constantly in the gg conformation. This discrepancy was attributed to an anomaly arising from insufficient equilibration in the 50 ns simulations [10]. Therefore, the extreme flexibility of Pn14 PS and disordered structure as compared to GBSIII PS suggested by this prior work could be a consequence of the increased flexibility of this dihedral in the AMBER force field as compared to the CHARMM force field. In the presence of competing computational models, experimental evidence is key. The DP2-Fab crystal structure provides evidence that a short strand can effectively bind to an antibody and that a helical conformation of the backbone is not necessary for antigen binding. In addition, we observed inter-residue NMR NOEs for the GBSIII PS that are consistent with our simulations.

The branches of the GBSIII and Pn14 PS both present common terminal glycan epitopes: 3-Sialyl-*N*-acetyllactosamine is present in human biofluids and is a common sequence terminating *N*- and *O*-glycans on the surface of all mammalian cells, while the shorter βdGal*p*(1→4)]βdGlc*p*NAc constitutes the ubiquitous LacNAc building block in mammalian N-linked protein glycans. In general, mimics of mammalian cell surface residues can subvert the immune system.

This work thus suggests a strategy for bacterial evasion of the host immune system: expression of a very flexible backbone that is shielded from the immune system by both its zig-zag conformation and hyper-mobility, combined with exposed, inflexible branches that present human-mimic epitopes. This strategy should be considered with other polysaccharides with similar backbones and human-like branch epitopes, where it is likely that the branch points will be key to immunogenicity.

In summary, this work demonstrates how small structural features such as side chains can alter the conformation of a polysaccharide by restricting rotation around backbone linkages. It also highlights the explanatory power of simulation for optimal antigen design and the development of effective conjugate vaccines.

## Abbreviations

The following abbreviations are used in this manuscript:

NMR	Nuclear Magnetic Resonance
PS	polysaccharide
RU	repeat unit

## Appendix A. Analysis of Glycosidic Linkages

The GBSIII glycosidic linkages can be conveniently classified into the three linkages which make up the common polymer backbone and the two glycosidic linkages which comprise the side chains. The contoured disaccharide ϕ, ψ PMF energy surfaces for disaccharides representing all the constituent linkages in GBSIII (Pn14 and the backbone being subsets) are shown in the left column of Figure A1. The three linkages which make up the common polymer backbone are shown in the top three rows and the two glycosidic linkages which comprise the side chains in the bottom two rows.

### Appendix A.1. β*d*GlcNAc(1→3)β*d*Gal

The βdGlcNAc(1→3)βdGal backbone linkage is flexible: the ϕ, ψ PMF (Figure A1a, left) shows a broad, shallow central well, encompassing the global energy minimum at ϕ, ψ = 54${}^{\circ }$, 16${}^{\circ }$, and a secondary syn-syn minimum at ϕ, ψ = 44${}^{\circ }$, −54${}^{\circ }$ with ΔG = 1 kcal·mol${}^{-1}$. This is compatible with the GBSIII DP2-Fab complex crystal structure [13], where this linkage has angles equivalent to ϕ, ψ = 35${}^{\circ }$, −9${}^{\circ }$ (indicated by ‘X’ on the PMF plot). In addition, the tertiary anti-ψ minimum is also relatively low in energy (ΔG = 1.5 kcal·mol${}^{-1}$), albeit with a high energy barrier to rotation of >6 kcal·mol${}^{-1}$.

The time series scatter plots for this linkage in GBSIII, Pn14 and the unbranched backbone show that all 6RU oligosaccharides explored the full range of linkage conformations in the central well, with the average ϕ,ψ value showing a shift to favor the secondary well in solution (Table A1). This is in agreement with the reported behaviour of this linkage in the sialyl Lewis X pentasaccharide (sLe${}^{X}$-5) for a 500 ns MD simulation at 37 °C in explicit water [45]. However, transitions to the anti-ψ conformation occurred rarely throughout the duration simulations. This is in contrast to sLe${}^{X}$-5, where the unconstrained terminal βdGal showed a significant population of the anti-ψ conformation. This difference is likely due to the more constrained environment in the oligosaccharides, as our βdGlcNAc(1→3)βdGal vacuum PMF is very similar to the energy map calculated for this linkage in sLe${}^{X}$-5. Indeed, the previous 5RU simulations of GBSIII and Pn14 by González-Outeiriño et al. showed an even smaller range of motion for the βdGlcNAc(1→3)βdGal linkage, encompassing only the vacuum global energy minimum (Table A1). This can be attributed to limited conformational sampling in the earlier 50 ns simulations, as transitions between the two minima in the central well occurred on a 20- to 50 ns time scale in our oligosaccharide simulations.

**Table A1:** Average values of the glycosidic dihedrals in degrees for the two middle repeating units of 6 RU of GBSIII and Pn14 and the unbranched backbone, including comparison with González-Outeiriño et al. [10] (standard deviations in parenthesis). * Note that the 1→6 linkage comprises three diehdrals.

	PMF	GBSIII	Pn14	Backbone
	ϕ, ψ, ω	ϕ, ψ, ω	ϕ, ψ, ω	
βdGlcNAc(1→3)βdGal	54, 16	45 (12), −22 (25)	45 (13), −20 (27)	44 (12), −23 (26)
González-Outeiriño et al.		42 (13), 2 (18)	41 (12), 1 (19)
βdGal(1→4)βdGlc	56, −1	47 (14), −14 (40)	49 (15), −14 (37)	49 (14), −17 (46)
González-Outeiriño et al.		47 (12), 8 (12)	43 (12), −7 (16)
βdGlc(1→6)βdGlcNAc *	26, 71	51 (10), −175 (11), −67 (8)	50 (12), −180 (18), −62 (26)	50 (15), −179 (22), −69 (30)
González-Outeiriño et al.		41 (10), −159 (22), −67 (9)	40 (14), −167 (31), −65 (10)
βdGal(1→4)βdGlcNAc	49, 1	50 (10), 0 (14)	50 (11), −2 (18)
González-Outeiriño et al.		39 (10), 5 (10)	43 (11), 5 (11)
αdNeu5Ac(2→3)βdGal	−66, 1	−70 (21), −8 (19)	-
González-Outeiriño et al.		−173 (14), −11 (11)

#### Appendix A.1.1. *β*dGal(1→4)*β*dGlc

The βdGal(1→4)βdGlc linkage is also flexible. The ϕ, ψ PMF (Figure A1b, right) has a single central syn-syn well with the global energy minimum at ϕ, ψ = 56${}^{\circ }$, −1${}^{\circ }$ and both a narrow anti-ϕ minimum (ΔG = 0.5 kcal·mol${}^{-1}$) and an anti-ψ minimum (ΔG = 3.6 kcal·mol${}^{-1}$). In the crystal structure of the six-sugar GBSIII epitope in complex with Fab, this linkage has angles equivalent to ϕ, ψ = −14${}^{\circ }$, 6${}^{\circ }$ and ϕ, ψ = 45${}^{\circ }$, −6${}^{\circ }$ (indicated by ‘X’s on the PMF plot). The 6RU simulations of GBSIII and Pn14 show sub-populations of anti-ψ conformations: transitions to this conformation are infrequent and require long simulation times to become apparent. Indeed, anti-ψ conformations of this linkage were not reported for the previous 50 ns simulation, which accounts for the smaller ψ standard deviation in the average value reported by González-Outeiriño et al. (Table A1).

#### Appendix A.1.2. *β*dGlc(1→6)*β*dGlcNAc

This linkage is discussed in detail in the main text.

### Appendix A.2. β*d*Gal(1→4)β*d*GlcNAc

Comparison of the PMF maps reveals that addition of a N-Acetyl group restricts the range of motion of the βdGal(1→4)βdGlcNAc linkage (Figure A1d, left) somewhat as compared to the βdGal(1→4)βdGlc linkage (Figure A1b, left). The energy of the anti-ψ minimum is slightly lowered and the anti-ϕ minimum raised. This is reflected in the narrower range of rotation in the simulations of the GBSIII and Pn14 6RU oligosaccharides (Figure A1d). Rotation to the *anti*-ψ orientation of this linkage is prevented by the proximity of the backbone βdGlc residue. In Pn14, the last RU which is not in contact with a branch has a significant population of the anti-ψ conformer. This is in agreement with simulations of both sLe${}^{X}$[20] and sLe${}^{X}$-5 [45], where the same linkage is constrained by the close proximity of the branching αlFuc.

### Appendix A.3. α*d*Neu5Ac(2→3)β*d*Gal

The PMF for the charged αdNeu5Ac(2→3)βdGal disaccharide PMF (Figure A1c, left) was computed in water and shows a very flexible linkage with multiple minima separated by low energy barriers. The conformation of this sialyl linkage is known to have no fixed fixed standard conformation and to be heavily dependent on the molecular environment [44]. Indeed, in contrast to the flexibility of the disaccharide, in our simulation of GBSIII 6RU, a *gauche* conformation predominates for this branching residue, with an average value of ϕ, ψ = −67${}^{\circ }$, −4${}^{\circ }$. The angle distribution for this linkage is consistent with previous MD simulations of sLe${}^{X}$[20] and sLe${}^{X}$-5 [45]. In contrast, González-Outeiriño et al. found that the (ϕ≈ −180${}^{\circ }$) *anti*-phi conformations predominated with the mean of ϕ, ψ = −173${}^{\circ }$, −11${}^{\circ }$ and a population ratio for the anti/gauche states of approximately 96:4 [10]. In the GBSIII DP2-Fab complex crystal structure [13], the unbound branch has angles equivalent to ϕ, ψ = −80${}^{\circ }$, −17${}^{\circ }$ (close to our values) the bound branch has ϕ, ψ = 54${}^{\circ }$, −4${}^{\circ }$ (indicated by ‘X’s on the PMF plot).
